# Discovery and development of Factor Xa inhibitors (2015–2022)

**DOI:** 10.3389/fphar.2023.1105880

**Published:** 2023-02-21

**Authors:** Wei Zheng, Xiaoqin Dai, Binyao Xu, Wei Tian, Jianyou Shi

**Affiliations:** ^1^ Pharmacy College, Chengdu University of Traditional Chinese Medicine, Chengdu, Sichuan, China; ^2^ Department of Pharmacy, Personalized Drug Therapy Key Laboratory of Sichuan Province, Sichuan Academy of Medical Sciences & Sichuan Provincial People's Hospital, School of Medicine, University of Electronic Science and Technology of China, Chengdu, China; ^3^ Department of Traditional Chinese Medicine, Sichuan Academy of Medical Sciences and Sichuan Provincial People’s Hospital, School of Medicine, University of Electronic Science and Technology of China, Chengdu, Sichuan, China; ^4^ Operations Management Department, Hospital of University of Electronic Science and Technology of China and Sichuan Provincial People’s Hospital, Chengdu Sichuan China School of Medicine, University of Electronic Science and Technology of China, Chengdu, Sichuan, China

**Keywords:** FXa, coagulation cascade, small molecule drug design, structure-activity relationship, anticoagulant activity

## Abstract

As a pathological coagulation process, thrombus can lead to many serious diseases, including ischemic stroke, acute myocardial infarction (AMI), acute coronary syndrome (ACS), and deep venous thrombosis (DVT). And anticoagulant drugs are one of the most effective ways to prevent and treat these diseases. Although macromolecular anticoagulant drugs such as low molecular weight heparins (LMWHs) are widely used in the clinic, their characteristics of requiring injectable use hinder their further promotion in the clinic, and the disadvantages of oral anticoagulant drugs, such as warfarin and dabigatran etexilate, which can easily cause bleeding adverse effects, are also not addressed. Factor Xa (FXa) has gained attention because it lies at the intersection of the coagulation cascade pathways, whereas subsequently introduced Factor Xa inhibitors such as rivaroxaban and apixaban, among others, have gained market popularity because of their high potency for anticoagulation and high specificity for Factor Xa when administered orally. But some of the drawbacks that these Factor Xa inhibitors have simultaneously such as fewer indications and the lack of an effective reversal drug when bleeding occurs are urgently addressed. The development of new Factor Xa inhibitors therefore becomes one means of addressing these questions. This article summarizes the small molecule Factor Xainhibitors developed from 2015 to 2022, classifies them according to their scaffolds, focuses on the analysis of their structure-activity relationships, and provides a brief assessment of them.

## 1 Introduction

In the normal physiological state of the human body, there are coagulation systems and fibrinolysis systems in the blood that resist each other. These two systems are in the process of dynamic equilibrium, which enables the blood to have a potential coagulation function and ensures that the blood is always in a fluid state. However, under the influence of certain factors (such as vascular endothelial injury, abnormal activation of the coagulation system, changes in blood flow, etc.), this dynamic balance is broken, so that blood coagulation or some formed components in the blood adhere to each other, the above process leads to the formation of thrombus (Holinstat, 2017).

As a common cardio-cerebrovascular disease, thromboembolism, in the process of development, can not only deepen the stenosis or occlusion of some vessels but also cause ischemia or infarction of the main organs of the body, thereby triggering physical dysfunction. Venous thromboembolism (VTE) (Duffy and Friedman, 2019), including deep vein thrombosis (DVT) and pulmonary embolism (PE), is common in cancer patients and patients undergoing total medullary or knee replacement surgery and is a cause of clinical death and disability. Thromboembolism is a major complication of atrial fibrillation (AF) and a leading cause of cerebrovascular accident. Acute coronary syndrome (ACS) is a group of clinical syndromes whose pathological basis is the rupture or erosion of coronary atherosclerotic plaque, followed by the formation of complete or incomplete occlusive thrombus. Symptoms of ACS include acute ST-segment elevation myocardial infarction (STEMI), acute non-ST-segment elevation myocardial infarction (NSTEMI), and unstable angina (UA), all of which are common clinical causes of disability or death. Clinically, these thromboembolic disorders seriously affect people’s lives and health because of their extreme morbidity and mortality (Larsen et al., 2016).

## 2 Coagulation process and anticoagulation therapy

### 2.1 Coagulation process

The physiological coagulation process of the human body is a cascade reaction process (Samama, 2011) in terms of initiating mechanism. This process can be divided into extrinsic and intrinsic approaches to its initiation. When the body’s blood vessels are damaged, the blood in the blood vessels is exposed to the surrounding collagen, which together with other factors causes the platelets in the blood to be activated. Due to the mediating action of von Willebrand factor (vWf) (Flood, 2015), platelets can adhere to collagen underneath the vascular endothelium, forming the initial thrombus. At the same time, due to vascular injury, the integrity of the vascular wall is destroyed, exposing tissue factor (TF) in the adventitia to the blood, thereby activating the extrinsic coagulation pathway (Wu et al., 2022). Subsequently, at the wound site, the formation of the TF-FVIIa (Grover and Mackman, 2018) complex promotes the activation of Factor X (FX) to Factor Xa (FXa), which also requires the help of Ca^2+^ and other cofactors. Ca^2+^ and FXa are key nodes in the coagulation cascade, leading to the conversion from prothrombin (FV) (Srivasatava et al., 2014) to FVa and accelerating the process. Thrombin converts fibrinogen into fibrin monomer using other factors (Ca^2^, FXIIIa, etc.), which combine to produce water-insoluble fibrin and then bind to a stable fibrin network. The adhesion of other blood cells (erythrocytes, platelets, etc.) through the fibrin mesh creates a stable thrombus (Wang et al., 2022). At the same time, thrombin promotes the activation of FXIII to promote platelet aggregation, further enhancing the thrombus’s strength.

The intrinsic coagulation pathway is more like the supplement and amplification of the extrinsic coagulation pathway, and both the contact activation system (CAS) and the kallikrein-kinin system (KKS) (Simberg et al., 2009) are involved in the coagulation process of the intrinsic coagulation pathway. CAS (Seo et al., 2019) is generally considered to be a passive defense system of the body against foreign substances, and its performance in the coagulation process is as follows: Conduction occurs when blood comes into contact with a negatively charged surface (Grover and Mackman, 2019) (such as some foreign proteins or materials, etc.), which activates the surface factor (FXII) to FXIIa (Ford et al., 2021). Subsequently, with the help of high molecular weight kininogen (HK) (Ponczek, 2021), FXIIa activates prekallikrein (PK) (Wang et al., 2022) to form plasma-type kallikrein (KAL) (Griesbacher et al., 2008). Bradykinin (BK) (Hofman et al., 2016), acting on Bradyldnin B2 Receptor (B2R) and Bradyldnin B1 Receptor (B1R) is an important pathway for KKS (Bekassy et al., 2022) to act, followed by a series of biological effects: Such as endothelium-dependent vasodilation, non-vascular smooth muscle contraction, etc. (Shariat-Madar et al., 2006). With the participation of CAS and KKS, FXIIa promotes the activation of FXI (Thomas et al., 2021), and FXIa promotes the formation of the FVIIIa-Ca^2+^ complex, which activates FIX with the help of the vWF factor. FIXa then promotes the production of FXa, which further enhances the entire coagulation process.

**FIGURE 1:**
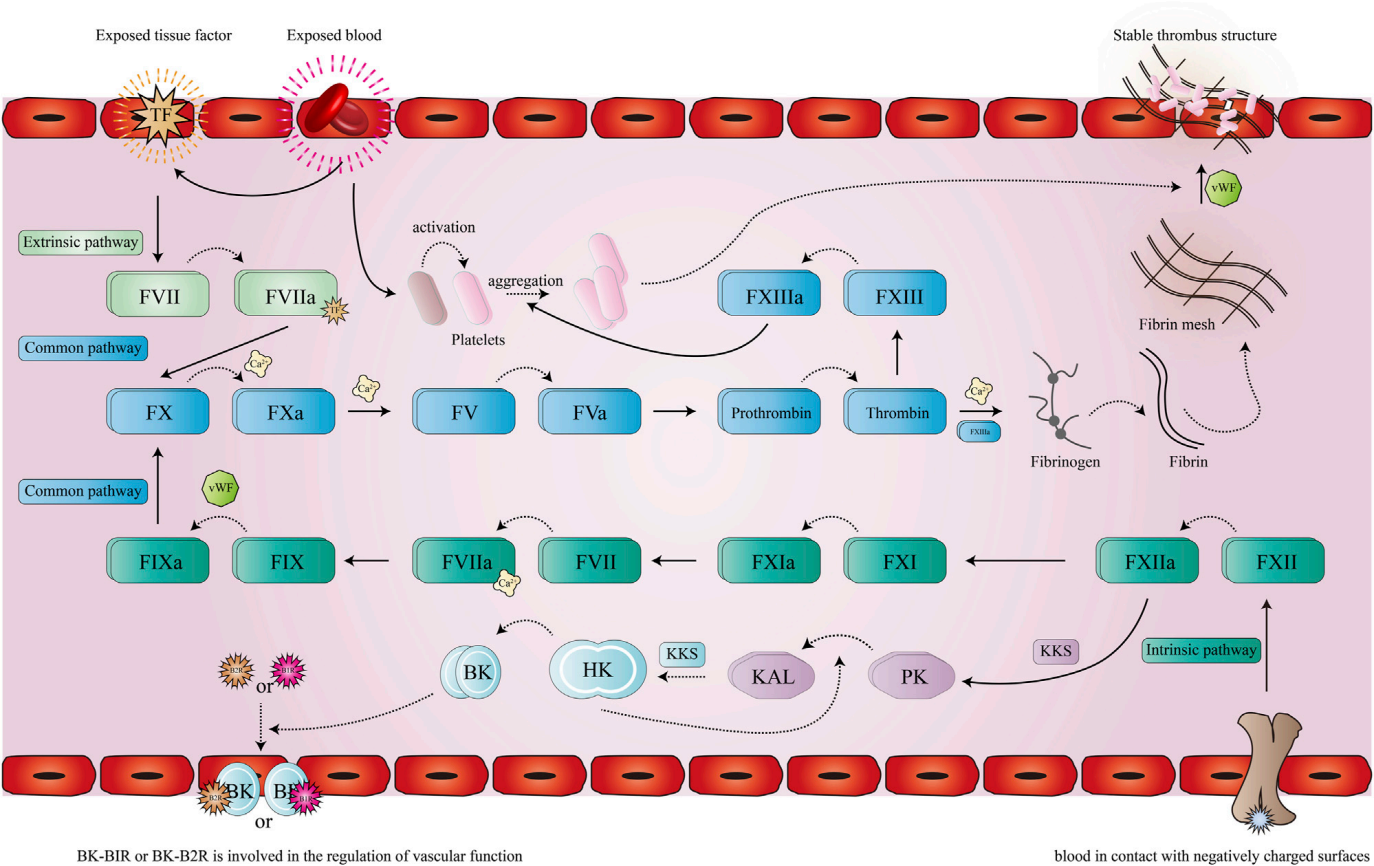
Pathway of coagulation. The solid line represents the promotion of a process, the dotted line represents the activation or the formation of a new product, after the extrinsic coagulation pathway (cyan) and the intrinsic coagulation pathway (green) are activated, the initial protein of the common pathway (blue) FX is activated and eventually leads to the formation of a stable thrombus.

In the coagulation cascade (Figure 1), FXa intersects the extrinsic and intrinsic coagulation pathways. It is a trypsin-like serine protease that plays an important role in coagulation (Bukowska et al., 2013). FXa can promote the activation of prothrombin to thrombin (Mackman et al., 2007), which is the main catalytic reaction in thrombus formation and wound closure (Ansell, 2007; Perzborn et al., 2011).

### 2.2 Anticoagulation therapy

Unfractionated heparin (UFH) (Derbalah et al., 2019) is a polymer of two polysaccharides with an average molecular weight of 12,000–15,000. UFH can act on a variety of clotting factors and has obtained a good blood thinning effect in clinical practice. However, it cannot be administered orally and the high risk of bleeding has hindered its clinical application. Low molecular weight heparins (LMWHs) (Hao et al., 2019) obtained by UFH degradation are safer macromolecular anticoagulants, which can inhibit two coagulation factors (FIIa and FXa) in time. LMWHs have a lower risk of bleeding, higher bioavailability, longer half-life and better pharmacokinetics properties than UFH. Therefore, LMWHs has almost replaced UFH in clinical practice, but it can only be injected, which brings inconvenience to its clinical promotion. Warfarin (Witt et al., 2016) is the earliest oral anticoagulant. It is a Vatamin K antagonist (VKA) (Piovella and Iosub, 2017), and although its oral nature has brought convenience to its clinical promotion, however, its narrow therapeutic window, unpredictable pharmacokinetic properties, and extensive food-drug and drug-drug interactions are the challenges in its clinical application. The subsequent application of thrombin (FIIa) (Stuart et al., 2020) inhibitors, such as Dabigatran etexilate (Paik, 2022), have improved the deficiencies of warfarin to a certain extent, however, it is easy to cause bleeding limits its clinical use.

**FIGURE 2:**
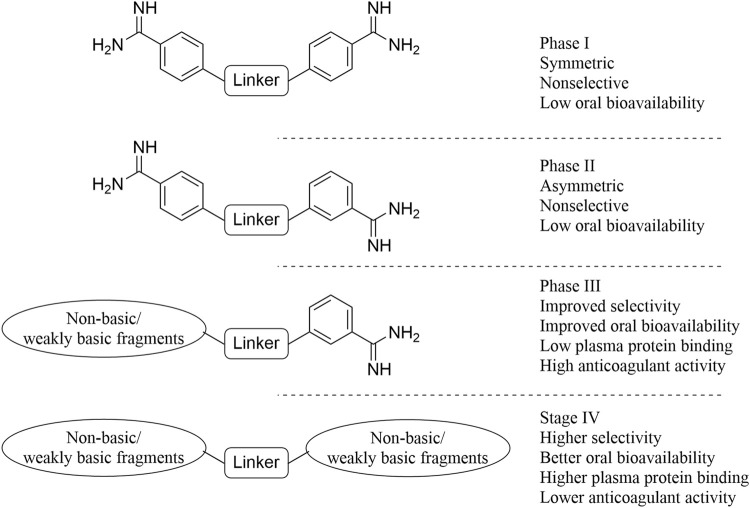
Development of FXa inhibitors.

The coagulation cascade (Camire, 2021) is a stepwise amplification process. It has been reported that for each molecule of Factor X activated, more than 1,000 thrombin molecules will be activated, and when FXa inhibitors play a role, they prevent further amplification of coagulation effect, thus effectively inhibiting thrombosis. According to the clinical efficacy of FXa inhibitors represented by Rivarxaban (Kvasnicka et al., 2017), FXa inhibitors have excellent anticoagulant activity and a wider therapeutic window than warfarin. Moreover, it has a larger anticoagulant range than dabigatran etexilate and does not cause hypercoagulable rebound state. Due to the high selectivity of FXa inhibitor compared to other anticoagulants, FXa inhibitors have a higher security profile and a lower risk of bleeding. However, because FXa inhibitors are small molecules, FXa inhibitors can be given conveniently versus large-molecule anticoagulants such as heparin. FXa inhibitors are classified as indirect or direct based on whether the synergistic effect of antithrombin III (AT III) (Lu et al., 2017) is needed or not. Indirect FXa inhibitors generally refer to heparins, and direct FXa inhibitors can be divided into peptide and synthetic types depending on their drug sources. However, due to the reasons of immunogenicity and low oral bioavailability, peptide FXa inhibitors have not attracted much research interest, while synthetic FXa inhibitors have attracted great attention in the area of medicinal chemistry. The development process is shown in Figure 2. Initially, when thrombin inhibitors were developed, it was found that some of these benzamidines exhibited moderate FXa inhibitory activity but were not selective for other coagulation factors in the coagulation pathway. Other studies have shown that molecular desymmetry can improve its activity and selectivity, but its oral bioavailability is mediocre. To improve oral bioavailability, one of the two strong basic bulking groups was replaced by a less basic or non-essential group. This strategy significantly improved the oral bioavailability and selectivity of the compound. Finally, on the basis of high activity, one of the remaining amidine motif segments was also replaced with weakly basic or non-basic fragments, and finally a small molecule direct inhibitor of FXa with good activity, high selectivity, and oral potency was found. However, after replacing the strong basic fragment of the molecule, the lipid solubility of the molecule was increased, which led to the increase of plasma protein binding of the compound, and the slightly reduced *in vitro* and *in vivo* anticoagulant activity. Further optimization of these parameters led to the discovery of compounds that have been on the market or in the clinical stage, such as Rivaroxaban, Apixaban, Edoxaban, Betrixaban, Omixaban etc.

## 3 Active site of FXa and its inhibitors

### 3.1 Active site of FXa

**FIGURE 3:**
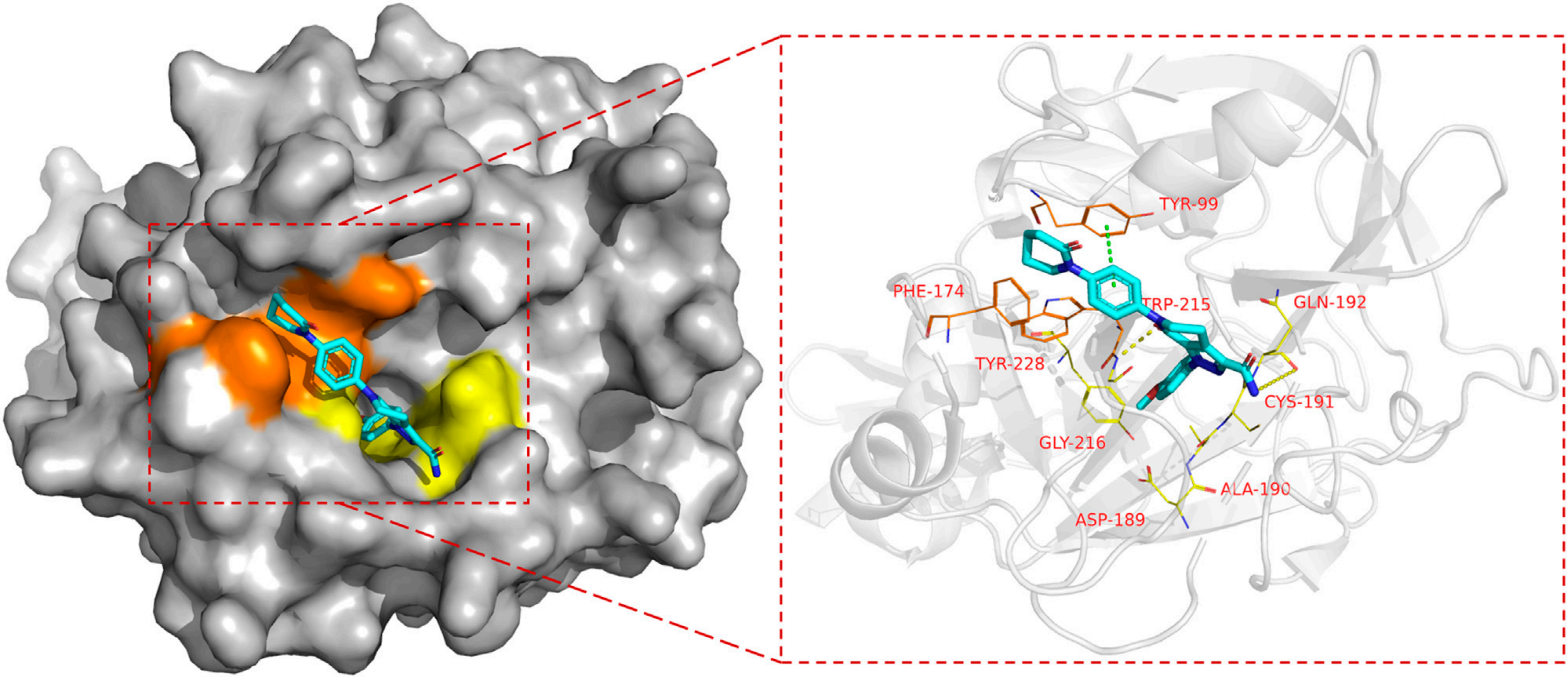
The binding mode of FXa, taking Apixaban as an example (PDB code: 2p16), the left side is the overall expression of Apixaban binding in FXa protein, the yellow area is the S1 pocket, the orange area is the S4 pocket. The right side is the expression of Apixaban in the active site of the receptor protein, where the yellow dotted line represents hydrogen bond, the green dotted line represents the Π bond, Amino acid residues in yellow form the S1 pocket, and amino acid residues in orange form the S4 pocket.

FXa is a vitamin K-dependent serine protease (Patel et al., 2016) composed of two amino acid chains (Hirayama et al., 2011) with different molecular weights. The active site of FXa is localized within the amino acid chain with greater molecular weight. According to Schechter and Berger (1968)'s binding site nomenclature for serine proteases and their substrates , the binding sites of FXa and its inhibitor (Kim et al., 2020) are located in the S1 pocket and S4 pocket. Although both S1 and S4 are strongly hydrophobic pockets, their composition is different. S1 (Roehrig et al., 2005) is a very narrow pocket, Trp215, Gly216 and Ala190, Cys191, Gln192 constitute the upper end of this pocket, and the bottom of the pocket is constituted by Asp189 and Tyr228, where small molecule inhibitors often form hydrogen bonds with acceptors. S4 (Maignan and Mikol, 2001) is an aromatic pocket, mainly composed of Tyr99, Phe174, and Trp215, this site is very different from other serine protease sites, so the Π force formed here also determines the small molecule inhibitor targeting (Figure 3).

### 3.2 FXa inhibitors from small molecules

All of the synthetic FXa inhibitors mentioned in this article that have been studied are in Table 1, with the structure appended to it for *in vitro* activity of FXa (indicated by IC_50_ values) and *in vitro* anticoagulant activity (indicated by 2 × PT values). In addition to the commonly used enzyme activity assays, Prothrombin time (PT) and Activated partial prothrombin time (APTT) assays should be added to determine the anticoagulant activity of FXa inhibitors *in vitro*.

**TABLE 1:** In the study of FXa inhibitors as well as relevant activity data (“Non” indicates that there is no corresponding data).

	No	R_1_	R_2_	R_3_	IC_50_	2 × PT	Ref
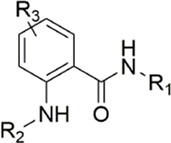	Compound 3	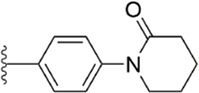	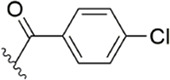	3-methyl	23 ± 8 nM	8.7 µ M	Xing et al. (2015)
	Compound 6	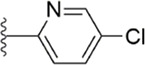	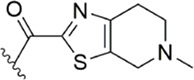	3-methyl	3.5 ± 1.0 nM	Non	Xing et al. (2018)
	Compound 7	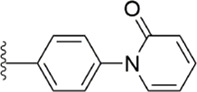	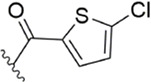	4-methoxy	25.0 nM	12.8 µ M	Wang et al. (2016a)
	Compound 8	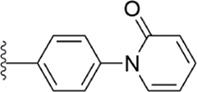	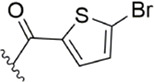	4-methoxy	13.4 nM	4.2 µ M	Huang et al. (2017)
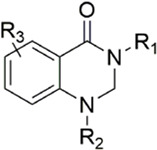	Compound 5	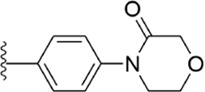	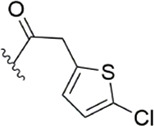	3-methoxy	21 ± 6 nM	12 µM	Xing et al. (2017)
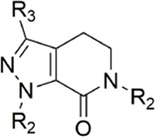	Compound 9	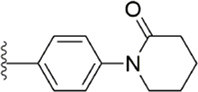	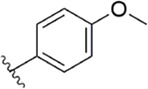	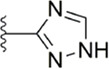	150 nM	0.5 µ M	Wang et al. (2016b)
	Compound 10	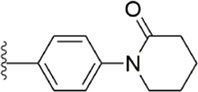	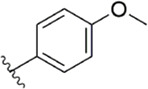	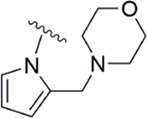	140 nM	0.5 µ M	Sun et al. (2017)
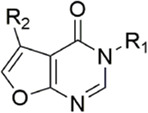	Compound 11	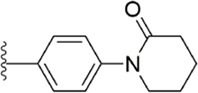	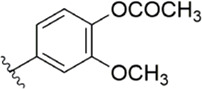	Non	13.0 nM	2.12 µ M	Yang et al. (2015a)
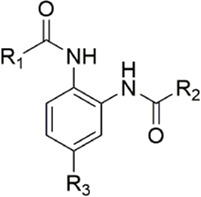	Compound 12	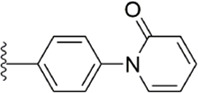	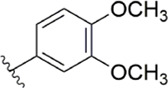	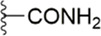	17.1 ± 0.9 nM	1.94 µ M	Yang et al. (2015b)
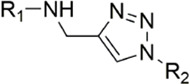	Compound 13	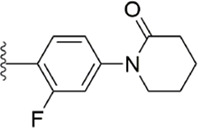	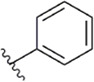	Non	102.1 nM	Non	Santana-Romo et al. (2020)
	Compound 14	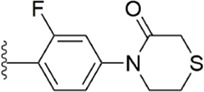	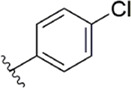	Non	67.9 nM	Non	Santana-Romo et al. (2020)
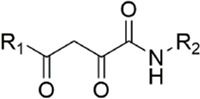	Compound 15	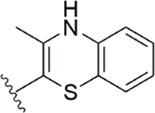	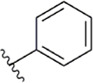	Non	2.02 nM	Non	Lagos et al. (2017)

#### 3.2.1 Anthranilate derivatives

As a common scaffolding, anthranilate has a broad spectrum of applications in drug development. At present, anthranilate derivatives have been used for anti-cancer (Gurulingappa et al., 2004; Labrie et al., 2006; Ott et al., 2016), anti-inflammatory (Shoji et al., 1988; Kukar et al., 2005; Narsinghani and Chaturvedi, 2006), soothing nerves (Englund et al., 2011; Joubert et al., 2011), and hypolipidemic (Roy et al., 2002; van Straten et al., 2002; Verma et al., 2015). Because of its good affinity for FXa targets, more and more researchers have designed it as the backbone of new FXa inhibitors. The summary of the structure-activity relationship for this scaffold is shown in Figure 3.

**FIGURE 4:**
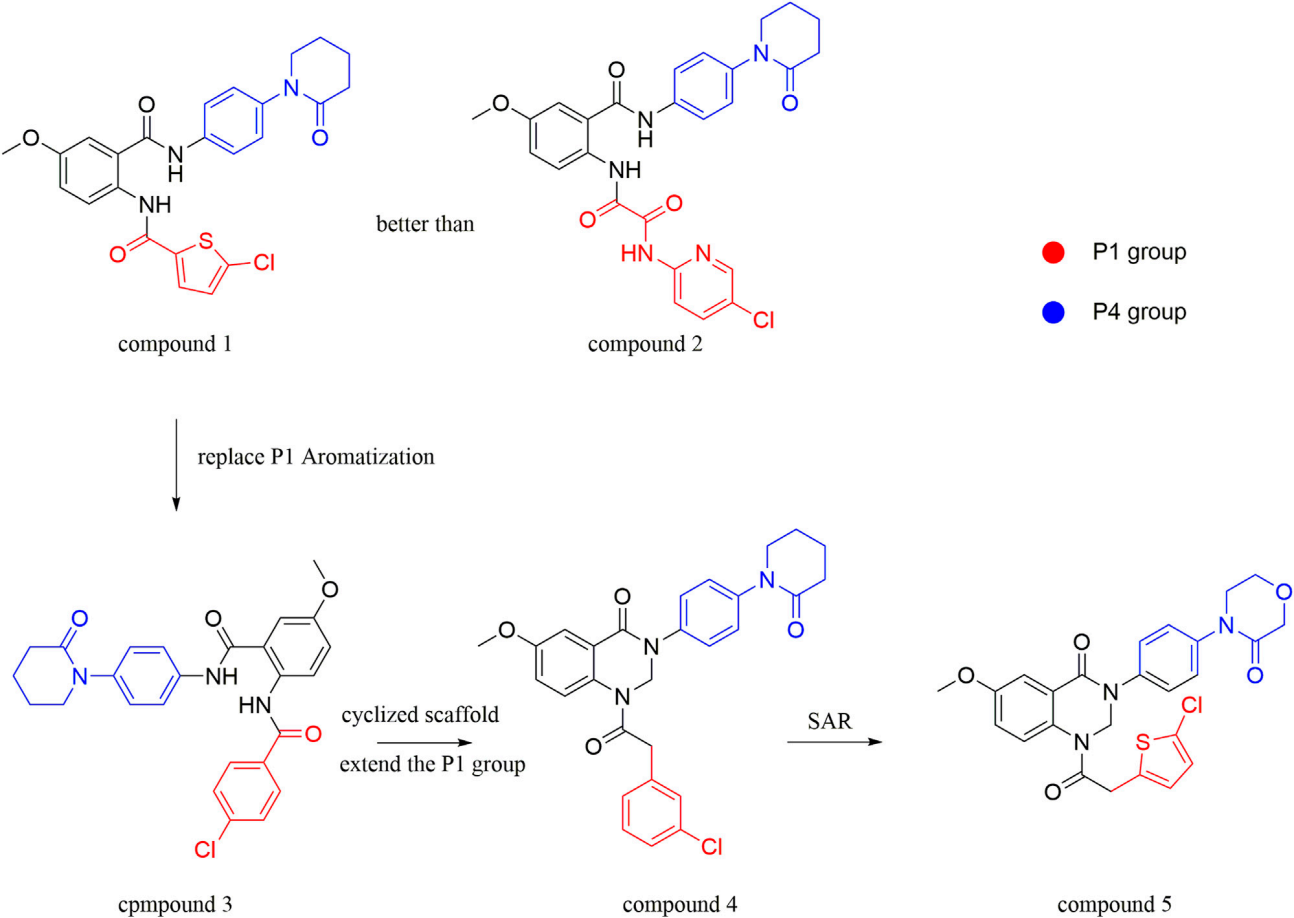
Design and structure of Compound 3 and Compound 5.

After the implementation of the compound docking strategy (Liu et al., 1994; Roehrig et al., 2005; Pinto et al., 2007; Ye et al., 2007; Corte et al., 2008; Yoshikawa et al., 2009; Anselm et al., 2010; Yuan et al., 2011), Xing et al. (2015) found two promising compounds: compound 1 and compound 2. From the docking situation, the phenyllactam structures shared by compound 1 and compound 2 have good complementary to the S4 pocket, which suggests the possibility of targeting this type of derivatives to FXa. Compound 2 was structurally modified. Following the activity verification, it was found that the activity of the compound 1 derivatives was better than that of the compound 2 derivatives. Zhang et al. estimated that the supplemental amide in the P1 part resulted in the low complementarity of the compound 2 derivatives in the S1 pocket, and this was verified in subsequent docking studies. The derivatives structure-activity relationship for compound 1 indicated that substitution at the para position of the phenyl ring P1 was the most active and required more volume in comparison with the substituents, where from the inhibitory activity against FXa, the inhibition ratio ranged from as high as chlorine > methoxy > fluorine. Among them, the *in vitro* activity of compound 3 (IC_50_ = 23 ± 8 nM) was the only one superior to that of compound 1 (Figure 4). Docking results also revealed that 4-chlorobenzamide exhibited excellent complement to the S1 hydrophobic pocket, in addition to forming a critical “Cl-P bond “with Tyr228, and its selectivity for FXa (compared to thrombin) was superior to that of Betrixaban but its anticoagulant activity was weaker than that of Betrixaban (2 × PT: 8.7 μM).

To further improve the selectivity and anticoagulant activity of the compound, Xing et al. (2017). Cyclized compound 4 and increased the length of the P1 moiety to obtain a series of derivatives based on compound 4. The docking results showed that the binding mode of compound 4 in both the S1 and S4 pockets was similar to that of compound 4. It is encouraging that compound 4 also kept the Cl-p key interaction in pocket S1. The advantage of morpholine-3-one on the aromatic pocket of S4 has been confirmed in the exploration of the structure-activity relationship, and the support of the key Cl-p bond formation by the length of the P1 part of compound 4 was again verified. In the substitution of P1 benzene, 5-chlorothiophene can better occupy the hydrophobic region of S1, which is also the key to the best activity of compound 5. Surprisingly, compound 5 was 3 times more selective for FXa (compared to thrombin) than Betrixaban, and its anticoagulant activity (2 × PT: 1.2 μM; 2 × aPTT: 0.6 μM) was also 2 times that of Betrixaban. In the rat arteriovenous shunt (AV-SHUNT) thrombosis model, compound 5 is similar to Betrixaban in reducing thrombus weight and prolonging bleeding time, which guarantees the safety of compound 5 (Figure 4).

**FIGURE 5:**
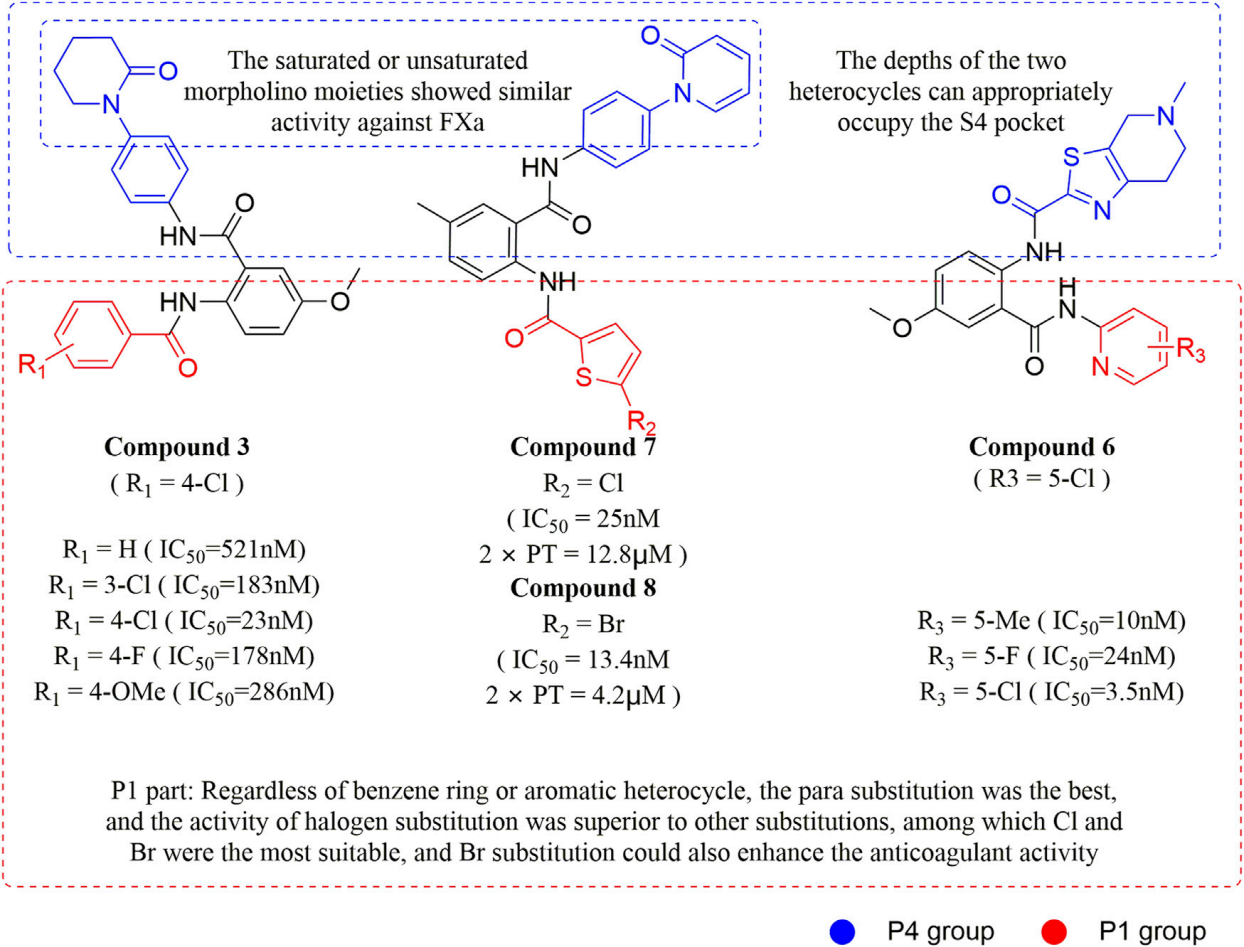
SAR of anthranilate derivatives.

Subsequently, Xing et al. (2018) reduced the basicity of the P4 moiety of Betrixaban and redesigned its P1 moiety to introduce new derivatives. The introduction of 6, 7-tetrahydrothiazolo [5,4-c] pyridine into the substituent of P4 significantly inhibited the inhibitory activity of FXa. On the basis of this P4 group, the influence of pyridine on the P1 moiety was superior to that of benzene and thiophene, but in agreement with their previous study, the chloro substitution on the aromatic ring as well as the position of the substitution was para - to the most favorable. Therefore, they reintroduced 5-chloropyridine into the substituent of P1 to obtain compound 6, which showed a 20-fold increase in the inhibitory activity against FXa (IC_50_ = 3.5 ± 1.0 nM) compared with compound 1. In the rat AV-SHUNT thrombus model, compound 6 can reduce more thrombus weight than Betrixaban at the same dose, and the bleeding time is consistent, which indicates that compound 6 can guarantee equal safety and is more efficient than Betrixaban. In addition, compound 6 has better selectivity for FXa (nanomolar level) than the micromolar level of inhibitory activity of other serine proteins. In the study of pharmacokinetics, compound 6 showed a good PK curve. It is worth mentioning that the bioavailability (F = 51.3%) of compound 6 in mice was almost twice that of Betrixaban (Figure 5).

Wang et al. (2016a) designed a series of anthranilate derivatives based on Rivaroxaban and Betrixaban. In terms of the substitution of the benzene portion of the scaffold, the substitution at the 5-position is superior to other positions, and introducing an electron-donating group here can bring a better effect on the inhibition of FXa. In addition, they increased the aromaticity of the P4 moiety and retained the characteristics of Rivaroxaban in the P1 moiety to obtain compound 7. Although the inhibitory effect of compound 7 on FXa (IC_50_ = 25.0 nM) did not improve much, the anticoagulant effect *in vitro* was enhanced (2 × PT (Human) = 12.8 μM), but it was still weaker than that of Rivaroxaban (2 × PT (Human) = 0.2 μM). Based on compound 7, Huang et al. (2017) replaced the chlorine in the P1 moiety with bromine, resulting in a stronger Br-p interaction in the S1 pocket, resulting in compound 8. Compared with compound 7, compound 8 showed increased FXa inhibitory (IC_50_ = 13.4 nM) and anticoagulant activity [2 × PT (Human) = 4.2 μM], suggesting a stronger interaction of bromo at the S1 pocket. The substitution of bromo at the P1 group by other compounds in this framework, such as compound 3 and compound 6, is worth further exploration. Therefore, based on this scaffold, enhancing the interaction force of compounds with the S1 pocket is a noteworthy direction (Figure 5).

#### 3.2.2 Pyrazolopyridone derivatives

**FIGURE 6:**
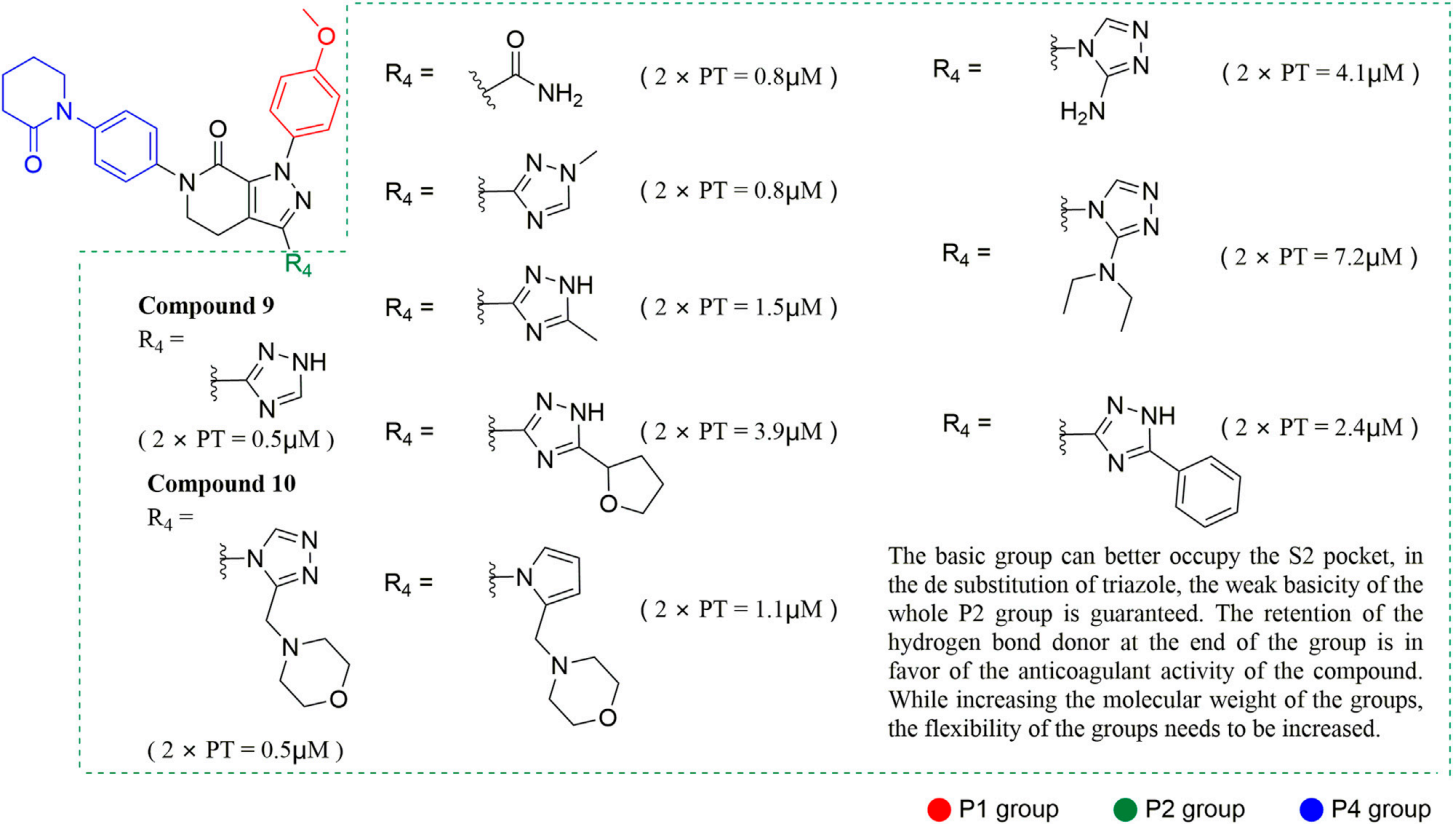
SAR of pyrazolopyridone derivatives.

Because of the complementarity of Apixaban to the FXa target, many researchers retain the backbone of its pyrazolopyridone to develop new FXa inhibitors. The pyrazole derivatives are also commonly used in the synthesis of numerous bactericidal drugs (Chen et al., 2000; Genin et al., 2000; Haque et al., 2002; Dunkel et al., 2003; Ehrenfreund et al., 2003). The summary of the structure-activity relationship for this scaffold is shown in Figure 6.

Wang et al. (2016b) made an in-depth focus on the S2 pocket of FXa while retaining the parent core of the pyrazolopyridone of Apixaban. After the introduction of different nitrogen-containing aromatic rings and amides, it was found that triazoles can produce more favorable hydrogen bonds to Glu146 in the S2 pocket, depending on the polarity and volume of the aromatic rings. The substitution of various saturated groups on triazoles can reduce the anticoagulant activity, which also verifies the above. Exploring P1 indicated that, compared to the strong interaction of halogens at the S1 pocket of anthranilate derivatives, due to the extended length and electric field of the pyrazolopyridone scaffold and its substituent groups at the junction of the S1 and S4 pockets. Due to the difference in strength, the methoxy group can induce the aromatic ring of the P1 moiety to form a stronger Π force due to its stronger electron donating ability and moderate size. Although this novel group of dihydroimidazole and tetrahydro pyrimidone was introduced to the P4 group, the anti-coagulant effect was even worse than that of piperidone, as indicated from the docking results with Phe174 in the S4 pocket Π The stronger force was the reason for the better *in vitro* anticoagulant activity of compound 9 (Figure 6). While *in vivo* anticoagulant activity, the inhibition rate of compound 9 on rat venous thrombus has been slightly higher than that of Apixaban.

Then Sun et al. (2017) explored the substitution of groups with different volumes on triazoles. For triazoles in the P2 part, they introduced different substituted benzene rings and various other saturated rings. From the results, the electron-donating ability of the substituents is favorable for the anticoagulant activity of the compounds, while more flexible groups seem to be more complementary to the S2 pocket. After introduction of a flexible morpho-methyl group into the P2 group, they obtained compound 10, which formed a new Π force in the S2 pocket of FXa and further enhanced the anticoagulant activity of these derivatives. The inhibitory activity of compound 10 on FXa *in vitro* was higher than that of Apixaban, and the anticoagulant activity *in vitro* was twice that of Apixaban. It is worth mentioning that the bioavailability of compound 10 is surprising, and its oral anticoagulant activity also surpasses that of Apixaban (Figure 6).

#### 3.2.3 Isoxazolopyrimidinones derivatives

The demonstration of the effectiveness of the monocyclic Isoxazoles in inhibiting FXa has even encouraged researchers to develop isoxazole FXa inhibitors, as Isoxazoles play important roles in cardiovascular disease, calcium regulation (Wittenberger, 1995; Johansen et al., 1998), and Alzheimer’s disease (Regine Riess et al., 1997).

**FIGURE 7:**
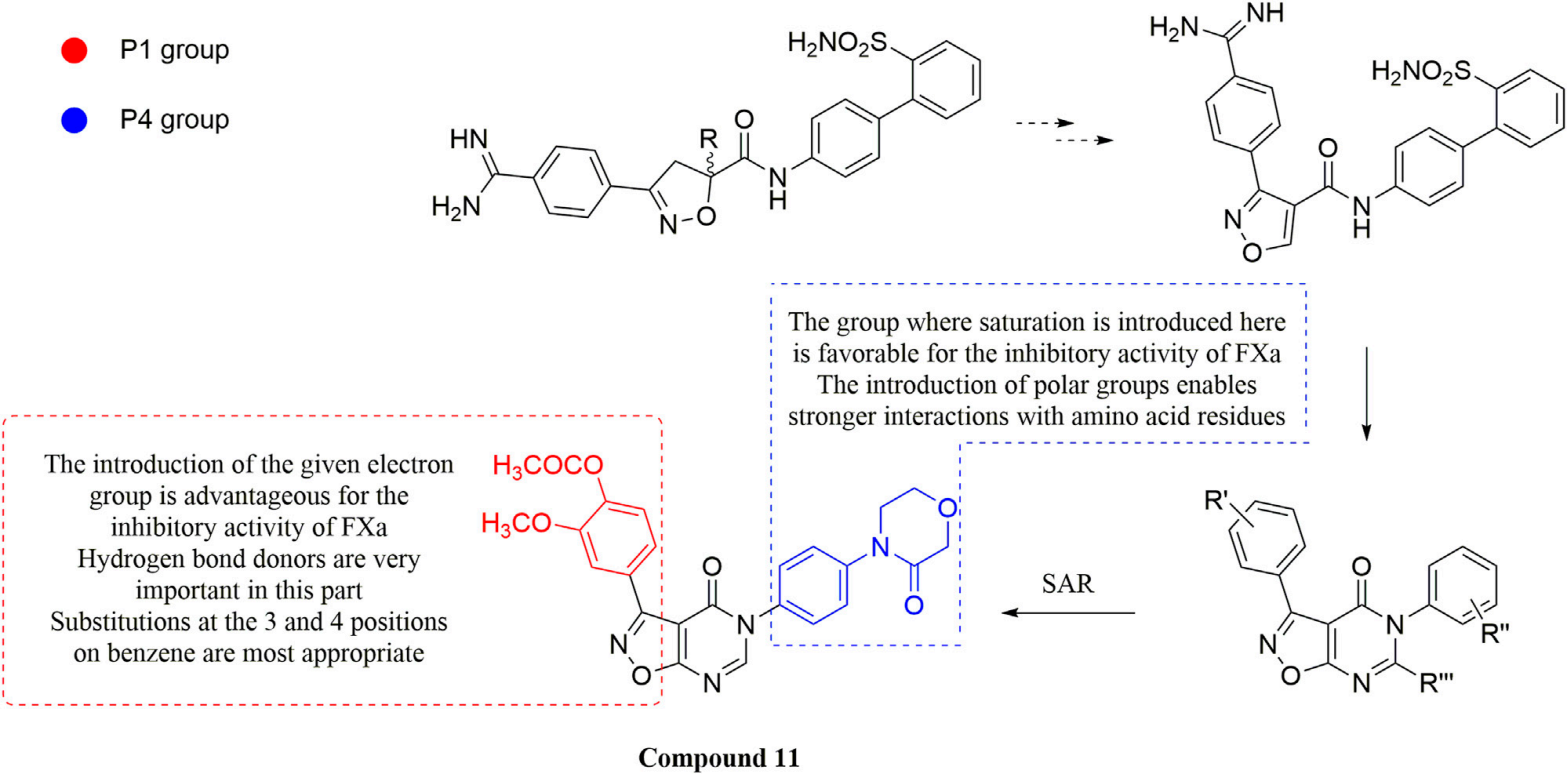
Design and SAR of compound 11.

Due to the reports of monocyclic isoxazoles (Quan et al., 1999a; Quan et al., 1999b; Wong et al., 2000a; Wong et al., 2000b) and bicyclic pyrazoles (Pinto et al., 2001; Lam et al., 2003; Pruitt et al., 2003; Pinto et al., 2006a; Pinto et al., 2006b; Pinto et al., 2007) FXa inhibitors and the confirmation of their effectiveness, Yang et al. (2015a). Decided to start with isoxazole scaffolds and reengineer the monocyclic isoxazole core (Krishna and Hayashi, 2000; Obe et al., 2002). They designed a series of derivatives with isoxazolopyrimidin as the original core, to obtain FXa inhibitors with a high affinity and hard to degrade. In the exploration of the P1 part, it was found that the introduction of the ester at the 4-position of the benzene ring can make the carbonyl group in it form a stronger intermolecular hydrogen bond with Gly216 of S1, and cooperate with the hydrogen bond of the methoxy group at the ortho position of its forest. The flexible substitution on the scaffold would destroy the complementarity of the derivative to the active site. The importance of morpholinone for the P4 part is also the same as in the previous study, and it is irreplaceable, in which the oxygen atom at position four acts as a hydrogen bond donor to enhance the complementarity of the compound with the S4 pocket, which is the other substituent (Such as benzene, pyridine, etc.) do not have. For the most active Compound 11 (Figure 7), its selectivity for FXa (IC_50_ = 13 nM) is 2000 times higher than that of serine proteases such as thrombin and trypsin, and the inhibition rate of FXa and anticoagulant activity *in vitro* are very close to Rivaroxaban.

#### 3.2.4 Diaminobenzamides derivatives

**FIGURE 8:**
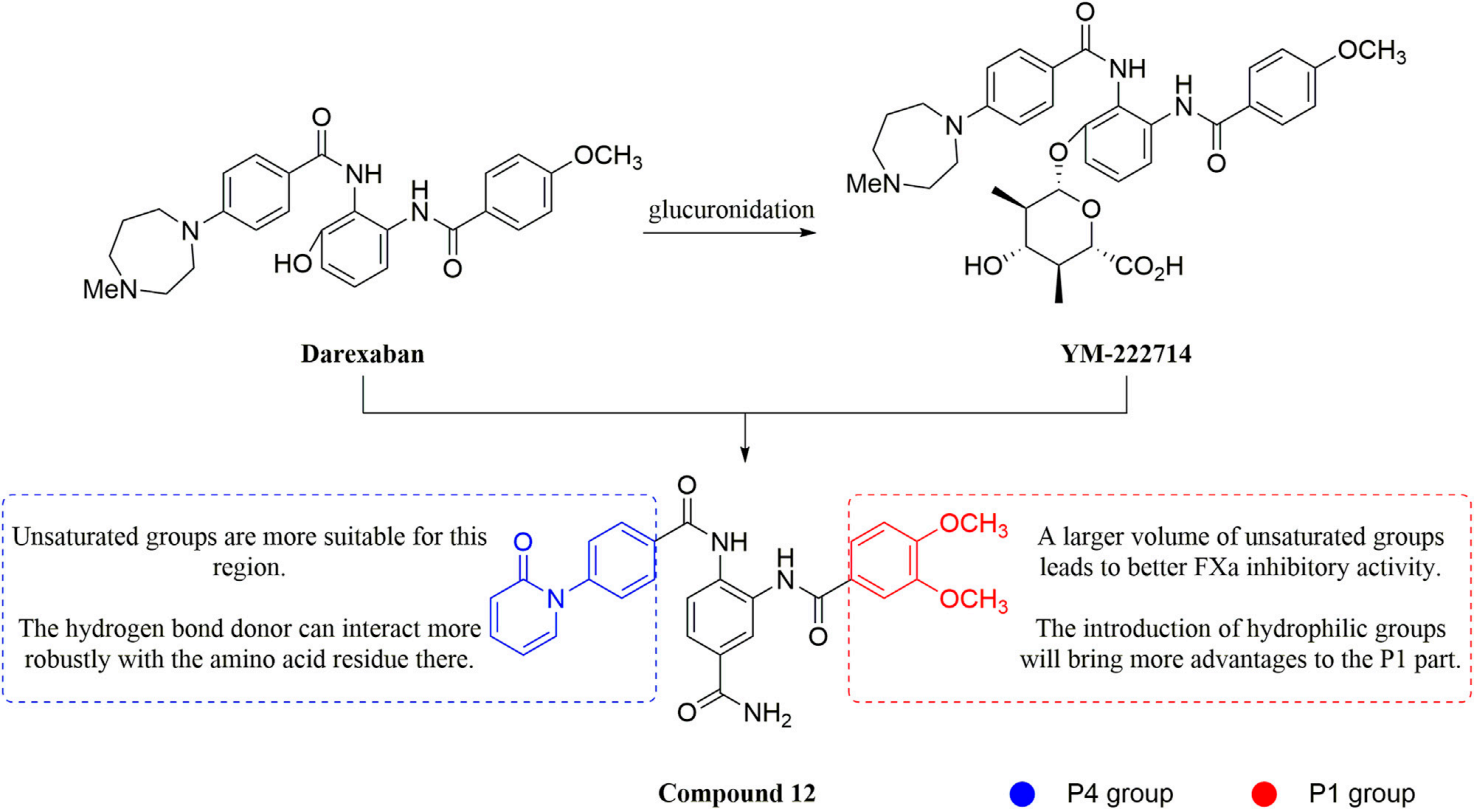
Design and SAR of diaminobenzamides derivatives and structure of compound 12.

Because the 1,2-phenylenediamine framework has many advantages such as less chirality, high affinity for FXa (Franciskovich et al., 2005; Koshio et al., 2005), and ease to get, meanwhile, FXa inhibitors with this structure also possess predictable pharmacokinetics (Kadokura et al., 2013a), small interactions with food (Kadokura et al., 2013b) and no inter drug interactions (Kadokura et al., 2014). Yang et al. (2015b) reported on the structural characteristics of Darexaban (Iwatsuki et al., 2011) with its glucuronic acid conjugate YM-222714 (Ishihara et al., 2014), Designed a series of FXa inhibitors with 3,4-diaminobenzidine backbone. They observed that when the volume of the aromatic ring of the P1 portion of the ligand was increased, the inhibitory activity of the compound would also increase and the increase in activity from the phenyl cycle would be due to other aromatic cycles. While, whereas in the structural optimization against P4 moiety, after contrast with lactams, pyridines of aromatic derivative showed better activity than the former, these results face to face P-force existed on the S4 pocket of FXa. In the substitution studies for the phenyl ring on the parent nucleus, the substitution at position five was greater than the substitution at position 4, and carboxamid substitution was a significant advantage over carboxylic acid and ester substitution. In further *in vivo* and *in vitro* FXa inhibitory activity screening, the hydrophilic group of P1 moiety was dominant. After a selective inhibition test, compound 12 (IC_50_ = 17.1 ± 0.9 nM) was the structure with the best comprehensive performance (Figure 8). Because of the excellent *in vitro* activity of compound 12, further *in vivo* activity testing should be necessary.

#### 3.2.5 Triazoles derivatives

**FIGURE 9:**
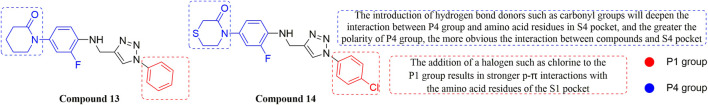
SRA of triazoles derivatives.

Because triazoles have become a recent research hotspot (Moura et al., 2016; Khalid et al., 2018) for antiplatelet activity, the Flavia C. Zacconi group (Santana-Romo et al., 2020) kept the PDB files of FXa ligand complexes containing phenoxymorpholine or phenoxypiperidine structures (Figure 9), from which to conduct virtual screening, and retained triazoles containing compounds for bioactivity evaluation. Inactivity assays on all structures, compound 13 (IC_50_ = 102.1 ± 0.14 nM) and compound 14 (IC_50_ = 67.92 ± 0.08 nM) show outstanding results at the vitro inhibitory activity. According to the subsequent assay, compound 13 was obtained to inhibit FXa by targeting both endogenous and exogenous pathways of coagulation factors, while compound 14 was obtained to inhibit FXa by both exogenous and common pathways. Whereas in the subsequent docking, compound 14 showed good complementarity in the binding site of FXa and benefited from the chlorine atom at its end, which could form a halogen bond with Aln190 in the active site, the thiomorpholine with triazole ring also provided several hydrogen bonds in the active site for binding. And the docking model compound 13 with key halogen bonds compared to compound 14 (Figure 9), the latter may account for its lower *in vitro* inhibitory activity.

#### 3.2.6 Dioxolamide derivatives

**FIGURE 10:**
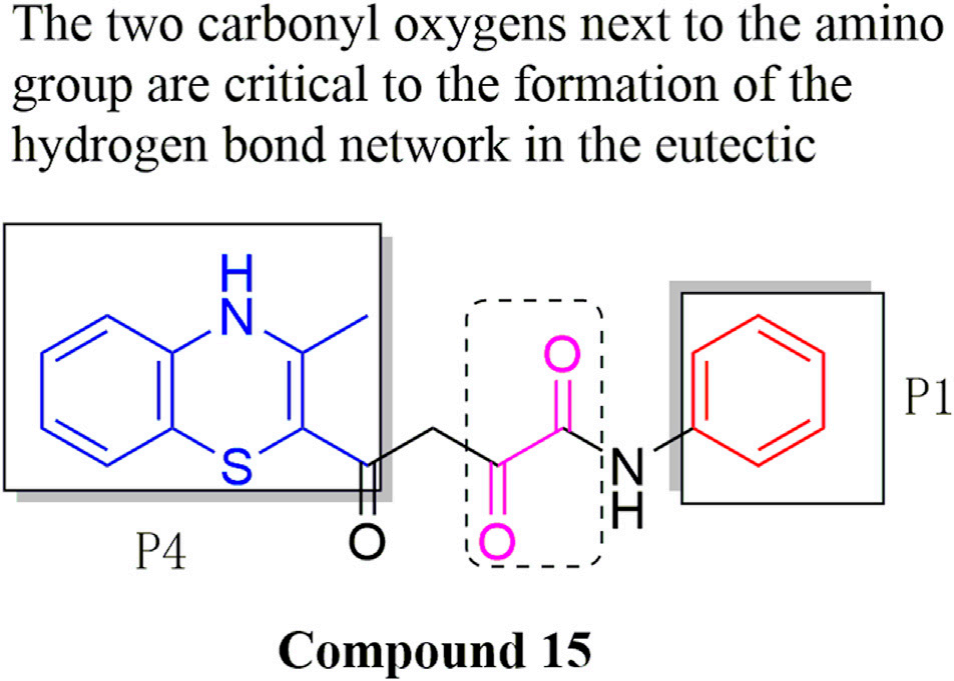
Structure of compound 15.

**FIGURE 11:**
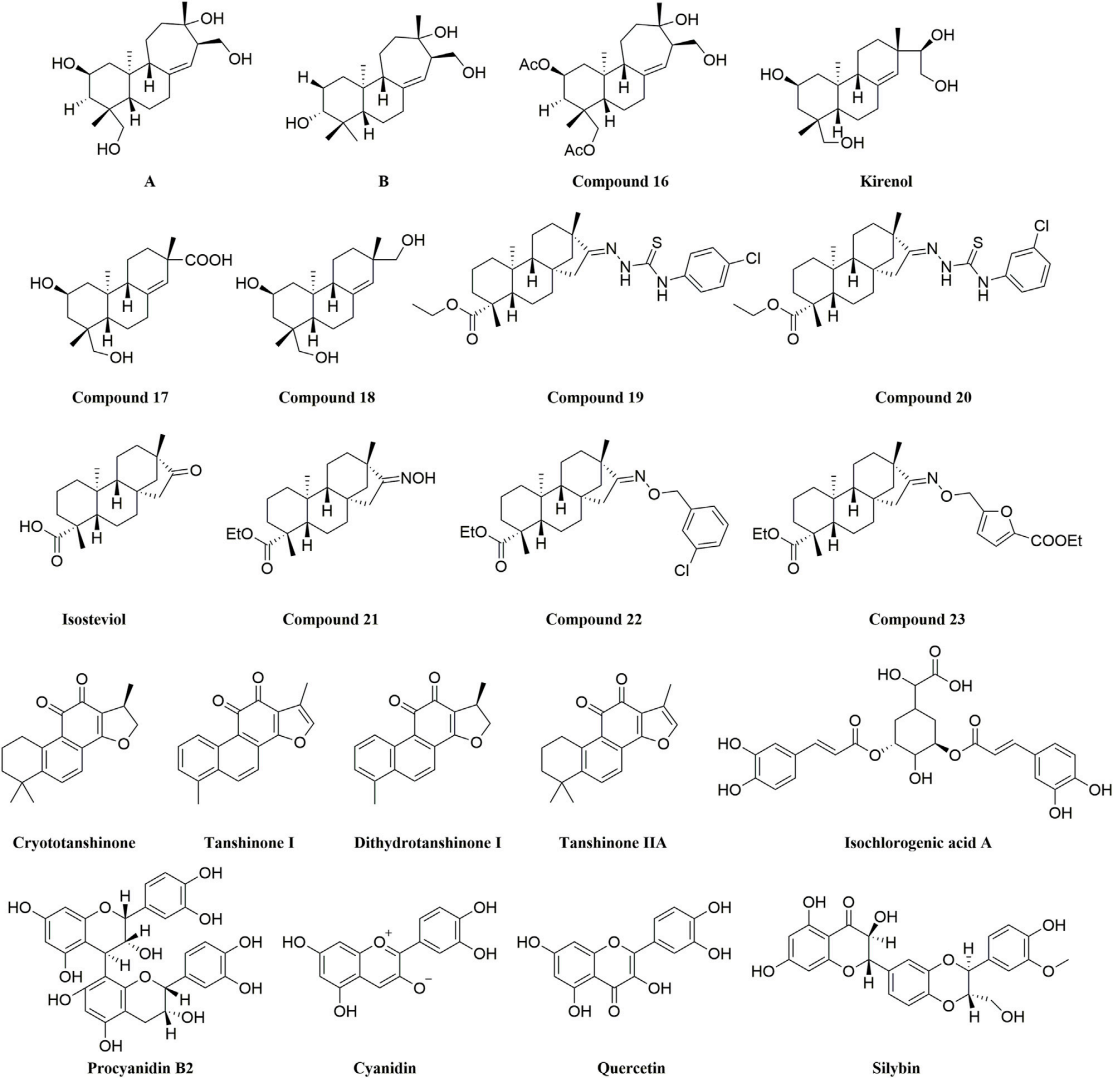
The structure of semisynthetic products and natural compounds.

Because of the important role that phenoxymorpholine and phenoxypiperidine structures (Khadse et al., 2018) exemplify in the binding process of Apixaban and Rivaroxaban to FXa targets. The Flavia C. Zacconi group (Lagos et al., 2017) retained small molecules that possessed the aforementioned structures to enable structure-based virtual screening in further bioactivity testing of the top-ranked compounds. Compound 15 (Figure 10) exhibited extremely excellent *in vitro* inhibitory activity (IC_50_ = 2.02 nM), which is already very close to that of the marketed Rivaroxaban (IC_50_ = 1.29 nM). In subsequent inhibitory rate tests against FXa, compound 15 get the inhibition rate of 58% at 10 μM class. In docking experiments, compound 15 exhibited a binding mode with FXa quite similar to that of Rivaroxaban, with its phenyl group attached to the amino group being the S1 pocket occupying the FXa binding site. And the amide group adjacent to it formed a hydrogen bond with the amino group of Gly216, and the two carbonyl oxygens next to it further formed a hydrogen bond network with Gly219 and Gly216, In contrast, the benzene ring, which is in phase with thiazine ring, occupied the S4 pocket of FXa the binding site well. If the X-ray diffraction co-crystal structure of compound 15 (Figure 11) and FXa can be obtained, the activity will have an excellent influence on the development of new FXa inhibitors, because it has a very different conformation from other conventional FXa inhibitors and has extremely high *in vitro* activity.

### 3.3 FXa inhibitors from natural products

Two strobe diterpenoid structures (A and B) were isolated from the aerial parts of Siegesbeckia by the Hongzheng Fu Group. (Wang et al., 2018) and will modify them structurally and evaluate their inhibitory effects on FXa to get steroidal diterpenoids with better anticoagulant activities. Among their semisynthetic 14 compounds, ent-norstrobane diterpenoids strombolis derivative (compound 16) performed a better FXa inhibitory activity (IC_50_ = 81 ± 11 nM). Likewise, (Wang et al., 2019) among the series of compounds obtained by modification with kirenol, a steroidal diterpenoid isolated in *Siegesbeckia Pubescens*, the FXa inhibitory activities of the oxidized compound 17 and the reduced compound 18 were the best (Figure 11).

Because steviol showed better activity against *Aspergillus flavus*. Group Fu (Shi et al., 2020) decided to use steviol as a starting material and introduce a triazole ring to its carboxyl terminus or a thiourea structure at the same position. And all of them were modified by introducing a phenyl ring at the terminal end of the structure, and all the derivatives were assayed for anti FXa activity with a hope to obtaining potential FXa inhibitors. From the data of the activity assay, the structure with triazole almost loses all inhibitory activity of FXa; Whereas in other derivatives, the introduction of electron-donating groups on the phenyl ring decreased the inhibitory activity, especially the introduction of a methyl group at the para position, directly inactivating the compound. In thiourea derivatives, the introduction of a chlorine atom on the phenyl ring enhanced the inhibitory activity. Especially at the para position (compound 19, IC_50_ = 152.78 ± 3.18 nM) and meta (compound 20, IC_50_ = 196.34 ± 5.37 nM) (Figure 11).

Subsequently, this group (Chen et al., 2019) screened the reported compounds (Chang et al., 2009; Wu et al., 2009; Zhu et al., 2013) and synthesized a series of isobutanol derivatives, in which: the introduction of ethyl acetate at 19-C would elevate the activity of the derivatives; The activity of introducing oximes at 16-c would be superior to that of introducing carbonyl and hydroxyl groups. So, they selected compound 21 (IC_50_ = 2.7 ± 0.3 μM) as the leading compound, and further modifications were finished. The group synthesized a series of oxime ether derivatives by the already mentioned oxime-based functional group with an inhibitory effect on FXa (Bauer et al., 2004; Huang et al., 2010), and subsequently, compound 21 was used to synthesize a series of derivatives that included P-P conjugated aromatic alkenyl. From the results of preliminary activity tests, the derivative of the m-chlorophenyl derivative with one more carbon in between (compound 22, Ki = 0.015 ± 0.002 μM) and the derivative of 5-ethyl acetate furan (compound 23, Ki = 0.028 ± 0.002 μM) showed the best activity to inhibit FXa and both of them better than leading compound (Figure 11).

Guo et al. screened the activity of various compounds extracted from *Radix Salvia Miltiorrhiza* in different solvent systems, and it was obvious that components extracted from EA had better inhibitory activity on FXa (inhibition rate up to 70%, 50 μg/mL) than others. Then, to visualize the chemical difference and predict the components responsible for inhibiting FXa, the principal component analysis (PCA) and orthogonal partial least squares discriminant analysis (OPLS-DA) were conducted on the MS data of Danshen fractions correlating with enzyme inhibitory activity. Cryptotanshinone, tanshinone I, dihydrotanshinone I and tanshinone II (Figure 11) were selected for further molecular docking. Contrary to the crystalline model of Rivaroxaban and FXa, tanshinone IIA was able to interact with the key amino acid residue Tyr99 to occupy the S4 pocket, and the other three compounds interacted with key residues such as Cys220 in the S1 pocket at various degrees, these may be the causes of the good inhibitory activity of FXa from the above compounds.

Then, Jian-Li Guo et al. used a similar method to test the activity of *Rhizoma Chuanxiong* extracts in various solvent systems. For FXa, compounds extracted from Butanol (BA) inhibited FXa by up to 80% (50 mg/mL). After modeling by the principal component analysis (PCA) and orthogonal partial least squares discriminate analysis (OPLS- DA), the specific marker compounds were predicted and identified. The most active compound identified was isochlorogenic acid A (IC_50_ = 0.56 mM). In subsequent molecular docking, isochlorogenic acid A (Figure 11) demonstrated a similar interaction with Rivaroxaban in the active site of FXa, these include π-interaction with the key Trp215, Cys220 in the S1 pocket, and hydrogen bonding with the key Tyr99 in the S4 pocket, it may be why isochlorogenic acid A is more active than other compounds.

From more than 20 polyphenolic compounds, Nowak et al. (Bijak et al., 2014) screened four flavonoid compounds that could significantly inhibit FXa, namely, procyanidin B2 (IC_50_ = 1.2 ± 0.2 μM), cyaniding (IC_50_ = 3 ± 0.2 μM), quercetin (IC_50_ = 5.5 ± 0.6 μM) and silybin (IC_50_ = 35 ± 3.5 μM) (Figure 11). Among them, procyanidin B2, cyaniding and quercetin all have a deep occupation to S1 pocket of FXa, particularly producing strong polar interactions with Asp189. However, silybin without dominant conformation has a low interaction in the S1 pocket (silybin only slightly interacts with Tyr228), as a result, the inhibitory activity of silybin for FXa is lower than procyanidin B2, cyanidin, quercetin and silybin. The pharmacophore model of procyanidin B2 (structurally similar to silybin) can provide guidance for further structure optimization of silybin, thereby further improving the inhibitory activity of silybin on FXa.

## 4 Discussion and prospect

By exploring p-benamidine’s FXa inhibitory activity, individuals can reduce the symmetry of this compound to enhance FXa inhibitory activity and selectivity, and replace the high basic group of the compound with low basic or non-basic groups can increase the bioavailability of the compound. Finally, FXa inhibitors with high selectivity and high orality are developed. As more and more FXa inhibitors are approved by the FDA to enter the market, people summarize the important amino acid residues of FXa receptor, which has guiding significance for the subsequent development and structural optimization of FXa inhibitors.

From the ligand-receptor co-crystal structure of FXa inhibitors that have been approved by the FDA, the most important structure of the patent drug targeting FXa protein is the structure of two pockets, S1 pocket and S4 pocket. Among them, Trp215, Gly216, Ala190, Cys191, and Gln192 together form the bottom and side of the narrow pocket of S1. From previous drug development experience, the hydrogen bond donor on the ligand can actively interact with the two amino acid residues Gln192 and Gly216 firmly. However, the hydrophobic pocket of S4 is mainly composed of Asp189, Tyr228, Tyr99, Phe174, and Trp215. Among them, Tyr99 is a very noteworthy amino acid residue, and the π interaction force formed by the halogen on the ligand is also the key to the inhibitory activity of the drug on FXa.

Compounds with anthranilate as the scaffold are easy to show good activity. From the molecular docking experiments of the research group of this type of compound, the carbonyl group (Gln192) and the amino group (Gly216) on the benzene ring may simply interact with the neighboring amino acid residues and undergo hydrogen bond interactions, so that the two-branched chains of the compound are more firmly stuck in the corresponding two pockets. In the benzene ring of the parent nucleus, the substitution at position 4 has a better effect than the substitution at its adjacent position. It may be because Gly219 can interact more closely to the substituent at this position, so the author thinks it can be appropriate. Add here some groups with active hydrogens to initiate possible interactions here.

The S4 pocket of the FXa site is a deeper and narrower hydrophobic pocket, so the P4 group of the compound has one more aromatic ring than the P1 group. For the P4 group, the benzene ring close to the parent nucleus can have a Π interaction with Tyr99 in an appropriate position. This interaction is very critical, which directly leads to the end of the P4 group extending as far as possible to the S4 Bottom of the pocket. The piperidone group is typically used as the end of the P4 group. Based on the results, the unsaturated group can often achieve better activity, which can be linked to the high hydrophilicity at the bottom of the S4 pocket.

In the P1 group of the compound, the carbonyl oxygen close to the parent nucleus can form a hydrogen bond with the water molecule near the S1 pocket, thereby forming a hydrogen binding network with neighbouring residues, which suggests that carbonyl, amino and other structures retention here is very important. The group at the end of P1 is very favorable for the occupation of the S1 pocket. The smaller five-membered aromatic ring can complement the S1 pocket. In the above compounds, due to the obvious aromatic thiophene, its substitution effect is best, and in a suitable position, Tyr99 can interact with P4. The terminal heterocycle forms the Π force.

In general, in the follow-up research and development of FXa inhibitors, relevant research groups should pay attention to the corresponding characteristics of the two pockets mentioned above, especially the combination of CADD, and try to design the structure of the compound into an appropriate L type, pay attention to the length of the two ends of the small molecule and its interaction with key amino acid residues.

Some plant extracts also have FXa inhibitory activity, such as those from *Salvia Radix Miltiorrhiza* and *Rhizoma Chuanxiong*. It is clear that natural products are a great treasure, and the establishment of rapid methods for activity prediction and composition identification is helpful to promote the application of natural products. From the above mentioned methods, the combination of LC-MS and OPLS-DA or the construction of analog pharmacophore model is feasible. From the previous experience in the development of macromolecules natural products, Schixator (Ding et al., 2022) and Lunathrombase (Gogoi et al., 2018) are representative natural macromolecules extracts, and it is also a highly feasible method to start from the animal or plant source to find other related compounds. For some small volume natural compounds, the Asp189 interaction in the S1 pocket is important for the anti-FXa activity of these compounds.

## 5 Conclusion

FXa is at the intersection of the common pathways of exogenous and endogenous anticoagulants, and inhibition of this coagulation factor can achieve excellent anticoagulant effects. As an established protein structure, the development of orally available small molecule inhibitors against FXa could be more convenient than the use of large molecule anticoagulants such as UFH and LMWHs in clinical practice. Because of the high specificity of the inhibitors developed for this target, FXa inhibitors have predictable, safer pharmacokinetics than first-generation oral anticoagulants, such as vitamin K antagonists, like warfarin, which means that they can be used clinically without the need for redundant bleeding monitoring. This is also an important reason for the concern about FXa.

This article summarized the small molecule. FXa inhibitors developed from 2015 to 2022. Among them, Pyrazolopyridone derivatives have demonstrated good anticoagulation activity in recent years, but their inhibitory activity on FXa is low, probably due to the further enhancement of the polarity of P2 moiety. Consequently, the compound’s inhibitory activity relative to other coagulation factors increased. This is not a good result in the development of FXa inhibitors, because it will lead to a low therapeutic window of the drug *in vivo*, and it is impossible to predict its pharmacokinetic properties, and it is necessary to monitor the possible bleeding in the body at all times after administration like Warfarin. Triazoles derivatives also have low inhibitory activity against FXa, but the absence of data on anticoagulant activity *in vitro* precludes speculation about the safety of this class of compounds. Indeed, imidazole-based compounds are commonly used in the development of kinase inhibitors like EGFR. Therefore, the development of these compounds in other applications is worthy of attention. Anthranilate derivatives seem to be a structure worthy of further development, so the *in vivo* anticoagulant activity and corresponding pharmacokinetic properties of anthranilate derivatives are worthy of immediate investigation. In addition to similar scaffolds, these compounds all have corresponding halogen groups. Therefore, it is worthwhile to explore and optimize the permeability of these compounds to the blood-brain barrier and their anticoagulant activity in the brain.
